# Missing clinical trial data: the evidence gap in primary data for potential COVID-19 drugs

**DOI:** 10.1186/s13063-021-05024-y

**Published:** 2021-01-15

**Authors:** Florence Rodgers, Toby Pepperrell, Sarai Keestra, Victoria Pilkington

**Affiliations:** 1 School of Medicine, Imperial College London, London, UK; 2 Amsterdam UMC, University of Amsterdam, Amsterdam, The Netherlands; 3 Department of Global Health & Development, London School of Hygiene and Tropical Medicine, London, UK; 4 Oxford University Clinical Academic Graduate School, Oxford, UK

**Keywords:** Clinical trial transparency, COVID-19 treatment, Coronavirus, Repurposed drugs, Safety information, Adverse events

## Abstract

**Background:**

Several drugs are being repurposed for the treatment of the coronavirus disease 2019 (COVID-19) pandemic based on in vitro or early clinical findings. As these drugs are being used in varied regimens and dosages, it is important to enable synthesis of existing safety data from clinical trials. However, availability of safety information is limited by a lack of timely reporting of overall clinical trial results on public registries or through academic publication. We aimed to analyse the evidence gap in this data by conducting a rapid review of results posting on ClinicalTrials.gov and in academic publications to quantify the number of trials missing results for drugs potentially being repurposed for COVID-19.

**Methods:**

ClinicalTrials.gov was searched for 19 drugs that have been identified as potential treatments for COVID-19. Relevant clinical trials for any prior indication were listed by identifier (NCT number) and checked for results and for timely result reporting (within 395 days of the primary completion date). Additionally, PubMed and Google Scholar were searched to identify publications of results not listed on the registry. A second, blinded search of 10% of trials was conducted to assess reviewer concordance.

**Results:**

Of 3754 completed trials, 1516 (40.4%) did not post results on ClinicalTrials.gov or in the academic literature. Tabular results were available on ClinicalTrials.gov for 1172 (31.2%) completed trials. A further 1066 (28.4%) had published results in the academic literature, but did not report results on ClinicalTrials.gov. Key drugs missing clinical trial results include hydroxychloroquine (37.0% completed trials unreported), favipiravir (77.8%) and lopinavir (40.5%).

**Conclusions:**

There is an important evidence gap for the safety of drugs being repurposed for COVID-19. This uncertainty could cause unnecessary additional morbidity and mortality during the pandemic. We recommend caution in experimental drug use for non-severe disease and urge clinical trial sponsors to report missing results retrospectively.

## Background

Coronavirus disease 2019 (COVID-19) is a pandemic infection caused by severe acute respiratory syndrome coronavirus 2 (SARS-CoV-2). Its global spread has been rapid and unprecedented, at the time of writing 54.1 million confirmed cases have been reported with 1.3 million deaths across 191 countries [1]. Currently, treatment options for COVID-19 are limited. However, several drugs developed for other indications have shown promising results against SARS-CoV-2 in vitro, in animal models, or in compassionate use trials [2, 3]. Many of these drugs are now being experimentally repurposed for COVID-19 or are undergoing clinical trials in humans [4]. Such candidates include nitazoxanide, remdesivir, favipiravir, lopinavir, darunavir, hydroxychloroquine, chloroquine and ivermectin amongst others [5].

Investigations into the efficacy of these experimental treatments for COVID-19 are ongoing. Meanwhile, in response to positive media coverage, some speculative rather than evidence-based, governments are stockpiling vast supplies of these treatments in anticipation of their licencing for COVID-19. Furthermore, national regulatory institutions, meant to safeguard against the unsafe use of drugs, are under increasing pressure to relax approval standards to accelerate market-entry for COVID-19 treatments. For example, on April 27, 2020, the U.S. Food and Drug Administration (FDA) approved the antimalarial drug hydroxychloroquine for emergency treatment of COVID-19 with unknown optimal dosage and duration of treatment [6]. However, the efficacy of hydroxychloroquine is still under question [7]. Furthermore, it is cardiotoxic at the higher doses which may be indicated for COVID-19, causing QT prolongation leading to ventricular tachycardia and death [8]. Care must be taken not to lose the rigorous safety standards usually stipulated for pharmaceuticals, even during a pandemic, to avoid unnecessary morbidity and mortality worldwide.

As many of these drugs have been used widely in other indications for years, there should be substantial information on safety, tolerability and pharmacokinetics available in the public domain, including public trial registries where trial sponsors are expected to upload summary results. According to the FDA Amendment Act 2007, the responsible party for applicable clinical trials registered on ClinicalTrials.gov must report results to a public register within 12 months of the primary completion date or in some cases risk a fine of $11,569 for every day results are delayed [9, 10]. Yet, whilst the International Committee of Medical Journal Editors (ICMJE) policy requires prospective registration of interventional studies on a WHO primary registry or on ClinicalTrials.gov, it does not currently require researchers to report summary results on these registries before academic publication [11]. Failure to share clinical trial results publicly can have repercussions for health and public expenditure, especially during a pandemic when there should be rapid sharing of results rather than the conventional academic publishing route.

The effects of poor clinical trial transparency were illustrated by the widespread stockpiling and prescription of oseltamivir (Tamiflu) during the H1N1 swine flu outbreak in 2009, despite a lack of evidence on safety and efficacy [12]. When the clinical study reports for this therapeutic were finally made publicly accessible, the benefits of the product turned out to be exaggerated and misrepresented in the journal publications compared to the underlying data. Following the same H1N1 outbreak, a novel vaccine (Pandemrix) was rapidly rolled out, but 7 years later, it emerged that the manufacturer had failed to disclose important internal pharmacovigilance data showing narcolepsy to be a rare side-effect [13]. Clinical trial transparency is therefore vital for maximising and unifying the sharing of data on efficacy as well as safety in one registry entry and is therefore vital for evidence-based medicine during this pandemic and beyond.

Public clinical trial registries are an important tool for transparent collaborative research. On these registries, safety and efficacy data can be uploaded freely, shortly after completion of the study, and protocol and data collection methods are still quality assessed [14, 15]. In contrast, academic publication may take significant periods of time and can be costly and selective, with time-intensive writing and review processes. Furthermore, clinical trial registry data is available free of charge and can be pooled without concern for silent outcome switching or publication bias [16–18]. Indeed, inclusion of unpublished study results from clinical trial registries in meta-analysis may provide important additional information on adverse events and more precise risk estimates than looking at journal publications alone [19]. Moreover, academic publications often fail to disclose all information on adverse events occurring during clinical trials; a systematic review comparing journal publications with related unpublished documents showed a lower number of adverse events in the published medical literature for 75% of included studies [20]. Care needs to be taken during the pandemic as interest in potential treatments for COVID-19 generates even greater incentive than normal for the publication of studies with positive results only. Without rapid sharing of datasets for drugs that may be repurposed for COVID-19, starting with the timely reporting of primary trial results, secondary analyses of safety data will be arduous and often incomplete [14, 21]. This may slow down the biomedical innovation process and could lead to preventable side effects occurring in vulnerable patients if safety information remains missing. As the pharmaceutical pipeline is accelerated to address the COVID-19 pandemic, enhancing clinical trial transparency is now more important than ever.

In this rapid review of ClinicalTrials.gov, we aimed to determine the scale of unpublished clinical trial results. This is important as a lack of primary data may hinder safety reviews of repurposed drugs for COVID-19. We reviewed the number of completed or terminated trials that have not reported due primary trial results for an extensive list of medications being repurposed for COVID-19. Specifically, we searched for any trial results from all previous indications for these drugs that have not been made available to the public, with no results published on either on the ClinicalTrial.gov registry or in the academic literature.

## Methods

We selected 19 potential treatments for coronavirus diseases based on information found in potential COVID-19 treatment reviews [5, 22, 23]. The drugs assessed were pirfenidone, hydroxychloroquine, azithromycin, favipiravir, oseltamivir, sarilumab, tocilizumab, remdesivir, leflunomide, interferon-alpha, lopinavir-ritonavir, darunavir-ritonavir, baloxavir marboxil, umifenovir, interferon-beta, sofosbuvir, nitazoxanide, APN01 and ivermectin (Table 1). Synonyms and chemical names for these drugs were taken from pubchem.ncbi.nlm.nih.gov (Appendix) [24].

**Table 1:** Trials registered to NCT for repurposed drugs, of which ongoing and suspended or withdrawn. Percentages are of all registered trials

Generic name	Total registered	Ongoing	Suspended/withdrawn	Completed
Pirfenidone	86	30 (34.9%)	2 (2.3%)	54 (62.8%)
Hydroxychloroquine sulfate	233	74 (31.8%)	5 (2.1%)	154 (66.1%)
Azithromycin	457	98 (21.4%)	16 (3.5%)	343 (75.1%)
Favipiravir	12	3 (25.0%)	0 (0.0%)	9 (75.0%)
Oseltamivir	127	18 (14.2%)	8 (6.3%)	101 (79.5%)
Sarilumab	37	19 (51.4%)	0 (0.0%)	18 (48.6%)
Tocilizumab (atlizumab)	360	84 (23.3%)	9 (2.5%)	267 (74.2%)
Remdesivir	12	11 (91.7%)	0 (0.0%)	1 (8.3)
Leflunomide	25	4 (16.0%)	1 (4.0%)	20 (80.0%)
Interferon-alpha	1161	71 (6.1%)	41 (3.5%)	1049 (90.4%)
Lopinavir/ritonavir	1015	66 (6.5%)	22 (2.2%)	927 (91.3%)
Darunavir/ritonavir	246	26 (10.6%)	4 (1.6%)	216 (87.8%)
Baloxavir marboxil	9	4 (44.4%)	0 (0.0%)	5 (55.6%)
Umifenovir	10	8 (80.0%)	0 (0.0%)	2 (20.0%)
Interferon-beta	343	43 (12.5%)	15 (4.4%)	285 (83.1%)
Sofosbuvir	261	75 (28.7%)	10 (3.8%)	176 (67.4%)
Nitazoxanide	57	8 (14.0%)	1 (1.8%)	48 (84.2%)
APN01 (ACE2 analogue)	1	0 (0.0%)	0 (0.0%)	1 (100.0%)
Ivermectin	101	19 (18.8%)	4 (4.0%)	78 (77.2%)
**Totals**	**4553**	**661 (14.5%)**	**138 (3.0%)**	**3754 (82.5%)**

The U.S. clinical trials registry (ClinicalTrials.gov) was searched for all trials that listed these drugs as an intervention. Results of the search were downloaded on 4 April 2020 [25]. Numbers of trials with and without results on the registry were recorded. For trials without results, trial status was determined (completed, ongoing, suspended, terminated or withdrawn). Trials listing ‘primary completion date’ in the future were counted as ongoing, if no primary completion date was available then the study completion date was used. Listed trial status was used to identify terminated, suspended and withdrawn trials.

**Fig. 1:**
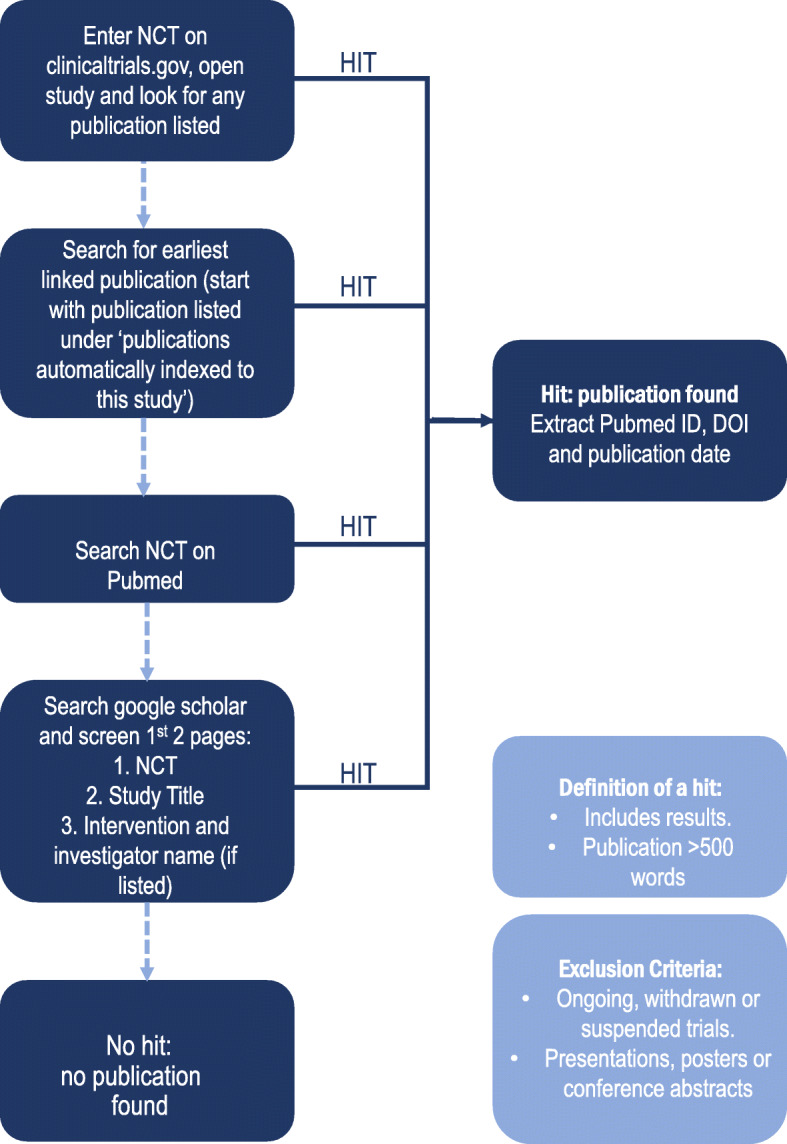
Flow chart depicting the methodology used to search for and identify relevant publications for each of the trials listed on CT.gov

For all trials without results on ClinicalTrials.gov, a three-step process was followed between 4 and 27 April 2020 to determine whether results were reported elsewhere through academic publication (Fig. 1).

1. Publications automatically indexed by clinical trial identifier (NCT number) on ClinicalTrials.gov were screened and included based on criteria below. If multiple publications were listed, the earliest dated publication was selected.
2. If results were not available on the registry, the NCT number was used to search and screen academic publications in PubMed.
3. If the PubMed search did not retrieve an academic publication, an additional search was conducted in Google Scholar using the following search terms in succession: clinical trial identifier; listed title; intervention name with primary investigator’s name. For each search, the first twenty results were screened.

If a publication did not include the clinical trial identifier, it was cross-referenced with the primary investigator, study design, intervention, and outcomes listed on ClinicalTrials.gov to assess relevance. We excluded publications that had fewer than 500 words, as well as conference abstracts, posters, presentations and non-English texts.

Publications of results were recorded by PMID, DOI and publication date. Trials with summary results on ClinicalTrials.gov combined with those with a journal publication gave a total number of trials with results where results were found in the public domain. This allowed approximation of registered trials without results.

Additionally, overdue trials were calculated as any completed trial with no result on the registry and a primary completion date before 18 April 2019, 395 days prior to final analysis (1 year + 30-day grace period). This is the standard outlined in the FDAAA 2007 and used as a reference throughout this study despite not all included trials being covered by the law [9]. This is also consistent with international ethical standards for timely results dissemination of 12 months set by the World Health Organisation [26].

A second blinded review was conducted by a different researcher on 10% of trials for each drug to check concordance between reviewers. The protocol during the second review remained unchanged and researchers were blinded to the results of the first review. A random number generator was used to select trials for second review. Concordance was assessed using simple percentage agreement as well as Cohen’s kappa to further analyse interrater reliability [27]. Results published between the dates of first and second review (29 April–9 May 2020) were not counted in this assessment.

## Results

**Fig. 2:**
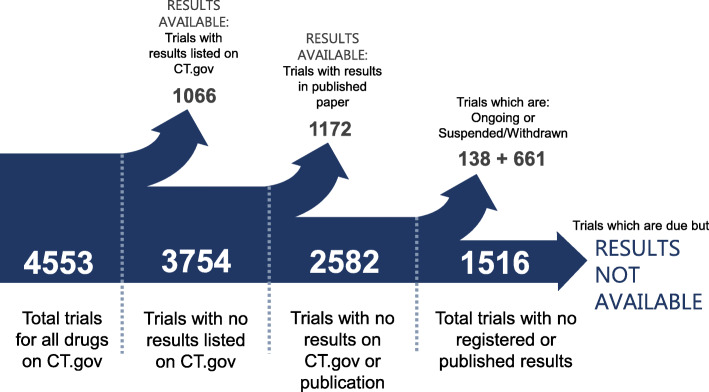
Flow diagram displaying the numbers of registered trials identified on CT.gov and the proportion of these which ultimately have no results available. The number of trials which had results available from various sources or had been withdrawn/suspended or trails for which results were not yet due are also shown

Nineteen drugs were screened, encompassing a total of 4553 clinical trials registered on ClinicalTrials.gov (Table 1). We excluded 799 of these trials, 661 of which were ongoing (primary completion date in the future) and 138 of which were suspended or withdrawn. Figure 2 shows the number of trials found on ClinicalTrials.gov, those excluded from this analysis, and the final results status of all included trials. All recorded percentages in text are in relation to the 3754 completed trials, seen in Table 2.

**Table 2:** Number of completed trials registered on ClinicalTrials.gov, of which results have been published on ClinicalTrials.gov or in the academic literature. Percentages are displayed as a proportion of all completed trials

Generic name	Completed	Results on NCT	Academic publication only	Results unreported
Pirfenidone	54	18 (33.3%)	20 (37.0%)	16 (29.6%)
Hydroxychloroquine	154	38 (24.7%)	59 (38.3%)	57 (37.0%)
Azithromycin	343	78 (22.7%)	119 (34.7%)	146 (42.6%)
Favipiravir	9	1 (11.1%)	1 (11.1%)	7 (77.8%)
Oseltamivir	101	38 (37.6%)	18 (17.8%)	45 (44.6%)
Sarilumab	18	13 (72.2%)	2 (11.1%)	3 (16.7%)
Tocilizumab (atlizumab)	267	125 (46.8%)	44 (16.5%)	98 (36.7%)
Remdesivir	1	0 (0.0%)	0 (0.0%)	1 (100%)
Leflunomide	20	5 (25.0%)	4 (20.0%)	11 (55.0%)
Interferon-alpha	1049	347 (33.1%)	301 (28.7%)	401 (38.2%)
Lopinavir/ritonavir	927	287 (31.0%)	265 (28.6%)	375 (40.5%)
Darunavir/ritonavir	216	73 (33.8%)	66 (30.6%)	77 (35.6%)
Baloxavir marboxil	5	2 (40.0%)	0 (0.0%)	3 (60.0%)
Umifenovir	2	0 (0.0%)	2 (100.0%)	0 (0.0%)
Interferon-beta	285	84 (29.5%)	84 (29.5%)	117 (41.1)
Sofosbuvir	176	36 (20.5%)	42 (23.9%)	98 (55.7%)
Nitazoxanide	48	11 (22.9%)	12 (25.0%)	25 (52.1%)
APN01 (ACE2 analogue)	1	0 (0.0%)	1 (100.0%)	0 (0.0%)
Ivermectin	78	16 (20.5%)	26 (33.3%)	36 (46.2%)
**Totals**	**3754**	**1172 (31.2%)**	**1066 (28.4%)**	**1516 (40.4%)**

**Fig. 3:**
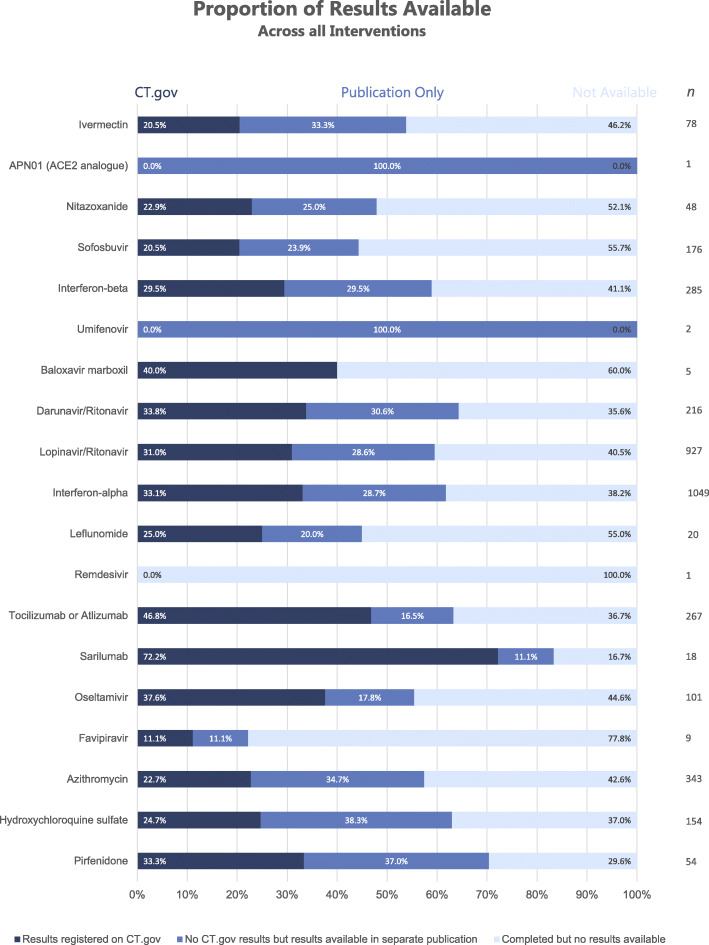
Bar chart displaying the proportion of trial results available across all potential COVID-19 therapies—categorised into those registered fully on CT.gov, those with results available in the academic literature only and those with no results available

In sum, our protocol revealed 2238 (59.6%) completed trials had published results either on the registry or in the academic literature (Table 2). Of these, 1172 (31.2%) completed trials had tabular results on ClinicalTrials.gov (Table 2). A further 1066 (28.4%) completed trials had results from the literature search, but did not report results on ClinicalTrials.gov (Table 2). Across the 19 drugs which may be repurposed for the treatment of COVID-19, 1516 (40.4%) of completed clinical trials listed on ClinicalTrials.gov were missing results. Figure 3 shows the proportions of trial results available on ClinicalTrials.gov, available in the literature, and those with no results available.

Of the 3754 completed studies, 2379 (63.4%) did not report results on ClinicalTrials.gov within of the 395-day timeframe mandated by the FDAAA 2007 and the WHO. Of these trials that did not report results on the NCT, 1008 (26.9%) had published results in the academic literature but failed to adhere to reporting best practices [9]. In the blinded second review of 341 (10%) trials, percentage agreement was 83.6%, whilst the kappa was 0.64 indicating ‘substantial agreement’ [27].

## Discussion

Of the completed clinical trials for existing drugs that may be repurposed for COVID-19, 40.4% did not report primary results, including safety data, on either ClinicalTrials.gov or through academic publication (Table 2). This shows a large gap in the evidence base regarding efficacy and adverse effects of these drugs, which may limit attempts to comprehensively review their safety before potential global distribution for the COVID-19 pandemic. As this review assesses reporting of primary results, which do not necessarily encompass safety data, the proportion of trials without safety information may be higher than the reported 40.4%. The 2238 (59.6%) completed studies with available results were comprised of 1172 (31.2%) with results on the registry and 1066 (28.4%) without results on the registry that had results from a standardised search of the literature (Table 2). Furthermore, 2379 (63.4%) studies without registry results had a primary completion date 395 days in the past and were therefore outside of the timeframe for results publication as mandated by the FDAAA 2007 and WHO best practice [9]. Although 1008 (26.9%) of these had already published results in academic literature, they still failed to upload their summary results on ClinicalTrials.gov. Not all trials included in this study are covered by the FDAAA 2007, but the 12-month deadline remains an important benchmark for good scientific practice [26]. With 40.4% of clinical trial results unavailable for potential COVID-19 treatments, the data for clinical decision making regarding the safety of these therapeutics is limited. If any potential treatments with an incomplete evidence base are used during the pandemic, even in compassionate use programmes, there is a risk of avoidable harm being done because of missing adverse safety data. An evidence gap was revealed for drugs which have had extensive media coverage in the context of COVID-19, including hydroxychloroquine (37.0% without results), favipiravir (77.8%) and lopinavir (40.5%) [28–30]. These drugs are currently used in COVID-19 patients and clinical trials across the globe, sometimes in novel regimens and doses that are much higher than those administered in the original trials [7, 31, 32]. Clinicians currently have few treatment options available, but with greater transparency and proactivity from trial sponsors regarding the posting of results, there would be less risk of unforeseen adverse outcomes, especially in the treatment of mild-moderate COVID-19 as in the PIONEER trial [31].

We recognise that there are sources of safety data beyond clinical trial registries, such as pharmacovigilance databases. However, these are not always publicly available. Additionally, there is often no obligation for all adverse events to be reported to pharmacovigilance registries, in contrast with clinical trial registries. Public health decision-makers, guideline developers, clinicians, and patients therefore rely on clinical trial registries, systematic reviews and meta-analyses to inform treatment decisions. Evidence gaps and publication bias therefore have the potential to influence clinical practice and drug usage worldwide, particularly in a treatment landscape as changeable as during the COVID-19 pandemic. Clinical decisions based on incomplete evidence can lead to avoidable morbidity and mortality, especially if unsafe drugs or ineffective treatments are given on a large scale. Sponsors and researchers alike carry an ethical responsibility to make results publicly available. They owe this to clinical trial participants, who consent to participate in research in order to contribute to scientific understanding and improved clinical practice [33].

Our study reveals an important evidence gap regarding existing pharmaceuticals potentially being repurposed for COVID-19. However, the proportion of studies with results available in the academic literature given here is an approximation and it is necessary to highlight several limitations to our study. Firstly, our trial population was limited only to those registered on ClinicalTrials.gov. Whilst ClinicalTrials.gov is the largest registry in the world, with over 340,000 registrations as of writing, and thereby an order of magnitude greater than the next largest registry, additional trials on these therapies may have been registered elsewhere and may not be captured in this study. However, it is unlikely that trials in other registries would report at a significantly different rate to ClinicalTrials.gov. As a second limitation, our strategy for locating publications included only those listed on ClinicalTrials.gov and identified through searches on PubMed and Google Scholar, open-access resources that should cover a majority of published clinical research. Including proprietary databases like Scopus or Ovid may have located some additional publications, yet we do not believe this would have substantially impacted our overall findings [34]. We are also aware that trials that were not registered in the first place, reported results in non-English language journals, or published without inclusion of the NCT number could potentially not have been captured by our methodology. Finally, searcher heterogeneity and difficulty identifying results publication in the academic literature limits accuracy of any manual publication search. However, our search strategy was standardised and produced a high level of agreement between assessors (83.6%) in a check of a 10% random sample. Furthermore, any discordance between reviewers only reveals the inherent difficulties in finding results for the drugs in question, especially if the trial identification number was omitted, in conflict with CONSORT standards [35].

Our findings add to the existing evidence of the dearth of accurate and timely clinical trial reporting on public registries. This analysis investigated clinical trials of existing drugs currently being considered for use for COVID-19. However, given the diversity of drug classes included in this report, findings are likely to be representative of many pharmaceuticals. This presents a major problem for researchers attempting to summarise safety and efficacy of such drugs by pooling existing trial data [36]. Since academic publications often summarise key findings only, secondary research efforts are impinged by the incomplete publishing of all trial outcomes. ClinicalTrials.gov, in contrast to academic publishing, provides a forum to share complete safety and efficacy data reports and facilitates consistent data reporting in a timely manner [14, 15]. Prior research has shown that results reported to ClincialTrials.gov were often more complete, especially for safety data, when compared to matched journal publications [16–18]. However, the availability of data depends on researchers registering trials and uploading results in a timely manner, within 12 months of the primary completion date.

The International Committee of Medical Journal Editors (ICMJE) and the editorial offices of medical journals could play an important role in improving the lack of timely results posting by demanding submission of a link to summary results on public registries before academic publication. Yet, this may mean that the publication bias of positive, ‘publishable’ results could trickle down to reporting on public registries as no such checks would exist for the reporting of negative results, which are less likely to be published in the first place. Furthermore, public funders and institutional publication funds could demand that trial sponsors post their results before allocating funding for academic (open-access) publication. These funding bodies could also deny individual sponsors funding if they have violated clinical trial reporting rules in the past [37], an option currently being considered by some UK funding bodies. At the very least, journals should conform to the CONSORT statement in ensuring that registry identification numbers are clearly indicated in the abstract, full-text and meta-data of published clinical trials in order promote discoverability and record linkage between registries and publications [35]. Finally, clinical trial sponsors, such as universities, hospitals, public research institutions and pharmaceutical companies, should themselves work towards improving their institutional clinical trial reporting performance by making use of available resources that provide detailed step-by-step instructions of this process [10]. Especially during the COVID-19 pandemic, it is of great importance that trials sponsors release summary results on these registries retrospectively to inform decision making around the safe usage of existing treatments being re-purposed for COVID-19.

## Conclusions

Overall, our findings reveal a significant evidence gap for drugs being repurposed for COVID-19. As a result important information on the safety of these treatments, that should have been reported with primary results, remains unknown. We suggest that this uncertainty could cause a large burden of extra morbidity in the global pandemic. We therefore recommend caution in experimental drug use for non-severe disease and urge trial sponsors to report missing results retrospectively. Medicine during the COVID-19 pandemic cannot be evidence-based if a large proportion of the evidence is missing

## Data Availability

The datasets generated and/or analysed during the current study are available on the U.S. National Library of Medicine (https://clinicaltrials.gov/ct2/home).

## Appendix

**Table 3:** Intervention clinicaltrials.gov search terms by generic name of drug

Generic name	Search terms
**Pirfenidone**	Pirfenidone OR Esbriet OR Pirespa OR Etuary
**Hydroxychloroquine sulfate**	Hydroxycholoroquine OR Plaquenil OR Hydroquin OR Axemal OR Dolquine or Quensyl or Quinoric
**Azithromycin**	Azithromycin OR zithromax OR AzaSite OR Zmax.
**Favipiravir**	Favipiravir OR Avigan OR T-705
**Oseltamivir**	Oseltamivir OR oseltamivir phosphate OR tamiflu OR GS-4104
**Sarilumab**	Surilumab OR Kevzara
**Tocilizumab (atlizumab)**	Actemra OR Tocilizumab OR Atlizumab
**Remdesivir**	Remdesivir OR GS-5734
**Leflunomide**	Leflunomide OR arava OR SU101
**Interferon-alpha**	IFN-A OR interferon alpha
**Lopinavir/ritonavir**	Kaletra OR lopinavir OR ritonavir or navir
**Darunavir/ritonavir**	Prezista OR Darunavir OR TMC 114.
**Baloxavir marboxil**	Baloxavir marboxil OR UNII-505CXM6OHG OR 505CXM6OHG OR 1985606-14-1 OR xofluza
**Umifenovir**	Umifenovir OR arbidol OR AR-1I9514 OR UNII-93M09WW4RU OR 131707-25-0
**Interferon-beta**	Interferon-Beta OR ampligen OR Betaseron OR betaferon OR BAY86-5046 OR Interferon beta-1b OR IFN-Beta
**Sofosbuvir**	SOFOSBUVIR OR PSI-7977 OR 1190307-88-0 OR SOVALDI OR GS-7977
**Nitazoxanide**	Nitazoxanide Oral Suspension OR Nitazoxanide OR Alinia OR 55981-09-4 OR Nitazoxamide OR Daxon
**APN01 (ACE2 analogue)**	APN01
**Ivermectin**	IVERMECTIN OR Ivermectin B1a OR Dihydroavermectin B1a OR 22,23-Dihydroavermectin B1a OR ivermectin H2B1a OR UNII-91Y2202OUW OR CHEBI:63941

## Abbreviations

COVID-19: Coronavirus disease 2019
CONSORT: Consolidated Standards of Reporting Trials
FDAAA2007: FDA Amendment Act 2007
ICMJE: International Committee of Medical Journal Editors
NCT Number: ClinicalTrials.gov identifier
PMID: PubMed ID
